# Antibacterial activity of traditional medicinal plants used by Haudenosaunee peoples of New York State

**DOI:** 10.1186/1472-6882-10-64

**Published:** 2010-11-06

**Authors:** Frank M Frey, Ryan Meyers

**Affiliations:** 1 Department of Biology, Colgate University, 13 Oak Drive, Hamilton NY 13346 USA

## Abstract

**Background:**

The evolution and spread of antibiotic resistance, as well as the evolution of new strains of disease causing agents, is of great concern to the global health community. Our ability to effectively treat disease is dependent on the development of new pharmaceuticals, and one potential source of novel drugs is traditional medicine. This study explores the antibacterial properties of plants used in Haudenosaunee traditional medicine. We tested the hypothesis that extracts from Haudenosaunee medicinal plants used to treat symptoms often caused by bacterial infection would show antibacterial properties in laboratory assays, and that these extracts would be more effective against moderately virulent bacteria than less virulent bacteria.

**Methods:**

After identification and harvesting, a total of 57 different aqueous extractions were made from 15 plant species. Nine plant species were used in Haudenosaunee medicines and six plant species, of which three are native to the region and three are introduced, were not used in traditional medicine. Antibacterial activity against mostly avirulent (*Escherichia coli, Streptococcus lactis*) and moderately virulent (*Salmonella typhimurium, Staphylococcus aureus*) microbes was inferred through replicate disc diffusion assays; and observed and statistically predicted MIC values were determined through replicate serial dilution assays.

**Results:**

Although there was not complete concordance between the traditional use of Haudenosaunee medicinal plants and antibacterial activity, our data support the hypothesis that the selection and use of these plants to treat disease was not random. In particular, four plant species exhibited antimicrobial properties as expected (*Achillea millefolium, Ipomoea pandurata, Hieracium pilosella*, and *Solidago canadensis*), with particularly strong effectiveness against *S. typhimurium*. In addition, extractions from two of the introduced species (*Hesperis matronalis *and *Rosa multiflora*) were effective against this pathogen.

**Conclusions:**

Our data suggest that further screening of plants used in traditional Haudenosaunee medicine is warranted, and we put forward several species for further investigation of activity against *S. typhimurium *(*A. millefolium, H. matronalis, I. pandurata, H. pilosella, R. multiflora, S. canadensis*).

## Background

North American Native American peoples used approximately 2,600 species of vascular plants in traditional medicines [1,2]. This figure represents roughly 10% of all vascular plant species in North America, and there is evidence that the choice of medicinal plants was non-random [3] and closely linked to phylogenetic relationships [4]. This bias in selectivity may speak to the efficacy of traditional remedies in the context of shared evolutionary descent (i.e., therapeutic compounds conserved among closely related plant species) much in the same way that certain classes of molecules are conserved among families such as the Apocynaceae (cardioactive glycosides), Caprifoliaceae (cyanogenic glycosides), Papaveraceae (alkaloids), and Solonaceae (glycoalkaloids) [5].

In Upstate New York, the Haudenosaunee (Iroquois) peoples used approximately 450 plant species in traditional medicine [6,7], which is similar in number to plant species used medicinally by neighboring First Nations Peoples in eastern Canada [8]. A number of studies have demonstrated concordance between selectivity of plants among First Nations Peoples to treat specific symptoms and demonstrated antibacterial, antifungal, and antiviral activity in the laboratory [9-16]. In this study, we use similar approaches to provide the first investigation of the antibacterial properties of plants used in Haudenosaunee traditional medicine.

We selected 15 plants for our investigation and screened them against four bacterial species. Four plants are considered among the most powerful medicines in traditional Haudenosaunee culture (*Achillea millefolium, Asclepias syriaca, Ipomoea pandurata, Malva neglecta*), serving as panacea remedies for a diversity of physical and spiritual ailments [6,7]. Five plants were used in Haudenosaunee medicines to treat a diversity of ailments (*Cimicifuga racemosa, Hieracium pilosella, Lycopodium digitatum, Ranunculus acris, Solidago canadensis*); although these were not considered among the approximately 150 most powerful medicines [6,7], they were well represented in regional Native American medicines [1,2]. In addition, we selected two native plant species (*Arabis glabra, Silene virginica*) and four introduced species (*Hesperis matronalis, Myosotis scorpioides, Rosa multiflora, Silene latifolia*) with no record of medicinal use among Haudenosaunee peoples [1,2,6,7].

We chose to test these plants against four bacterial species that vary with respect to their disease modality and virulence (*Escherichia coli, Salmonella typhimurium *[*Salmonella enterica *subspecies *enterica *serovar Typhimurium], *Staphylococcus aureus, Streptococcus *[*Lactococcus*] *lactis*). *Streptococcus lactis *is a gram-positive species used extensively in the dairy industry and regarded as an avirulent opportunistic human pathogen [17-19]; similarly, *E. coli *is a gram-negative human enteric species that is generally avirulent but sometimes causes weakly virulent gastroenteritis and urinary tract infections [19]. *Salmonella typhimurium *is a gram-negative pathogen that most commonly causes enterocolitis, and *S. aureus *is a gram-positive pathogen that most commonly causes abscess, food poisoning, and toxic shock syndrome; both are considered to be more virulent than *S. lactis *and *E. coli *[19].

Broadly, we hypothesized that we would find congruity between the use of particular plant species to treat specific symptoms by Haudenosaunee peoples and demonstrated antibacterial properties. Specifically, we hypothesized that plants used to treat symptoms possibly associated with bacterial pathogens (e.g., antipyretics, antidiarrheals, antiemetics, broad gastrointestinal treatments, dermatological ailments, etc.) would show more antibacterial activity than those plants used to treat other symptoms (e.g., analgesics, blood medicines, contraceptives, etc.). Additionally, we hypothesized that the greatest antibacterial effects would be observed in the more moderately virulent pathogens (*S. typhimurium *and *S. aureus*) compared to the less virulent pathogens (*S. lactis *and *E. coli*).

## Methods

### Plant collection and extract preparation

Plants used in traditional medicine by the Haudenosaunee (Iroquois) Confederacy in Madison County, NY USA were researched and located in field surveys (Table 1). Voucher specimens were prepared and submitted to the George R. Cooley Herbarium (GRCH). With the exception of *Lycopodium digitatum *in which only leaf material was collected, roots, shoots, leaves, and flowers of all plant species were collected and transported to the laboratory on ice where they were prepared immediately for extraction. At least six individual plants were collected for each species and plant material was pooled prior to making extractions. Four grams of pooled plant material was rinsed with sterile water (with the exception of flowers), cut into thin sections using a sterile razor blade and combined with 40 mL of sterile water. The mixture was homogenized using a sterile mortar and pestle, and the resulting solution was placed in a sterile 50 mL centrifuge tube, gently shaken at 4°C overnight, and centrifuged at 15,000 × g for 10 minutes to pellet solids. The supernatant was then withdrawn, placed in a sterile 25 mL scintillation vial, and stored at 4°C. Extracts were tested using the disk diffusion technique within 48 hours of preparation and tested using the 96-well plate assay within a week of collection. A total of 57 different extracts were created from these 15 plants, and at least two replicate extractions were made for each. The initial concentration of extracts used in all assays was 100 mg fresh material to 1 mL of water.

**Table 1:** Plant species utilized in this study and Haudenosaunee medicinal properties.

Species	Common name	**Medicinal Properties**^**a, b**^
"Powerful" Haudenosaunee medicinal plants^c^		
*Achillea millefolium*	Common Yarrow	Analgesic (LV, RT), Antidiarrheal (PL), Antiemetic (LV, ST), Antihelmintic (LV), Antipyretic (PL), Antirheumatic (PL), Blood (PL), Gastrointestinal (PL), Panacea drug (LV), Venereal Disease (PL)
*Asclepias syriaca*	Common Milkweed	Circulation (RT, PL), Dermatological (ST), Gastrointestinal (LV), Gynecological (PL)
*Ipomoea pandurata*	Man of the Earth	Analgesic (RT, PL), Blood (RT, LV, ST), Cough (RT), Gastrointestinal (RT, PL), Liver (PL), Panacea (PL), Witchcraft (PL)
*Malva neglecta*	Common Mallow	Analgesic (PL), Dermatological (PL), Gastrointestinal (PL), Orthopedic (PL)
Other Haudenosaunee medicinal plants		
*Cimicifuga racemosa*	Black bugbane	Antirheumatic (RT, PL), Blood (RT), Orthopedic (LV)
*Hieracium pilosella*	Mouseear hawkweed	Antidiarrheal (PL)
*Lycopodium digitatum*	Fan clubmoss	Anticonvulsive (PL), Diuretic (PL), Reproductive (PL)
*Ranunculus acris*	Tall Buttercup	Analgesic (PL), Antidiarrheal (RT), Blood (PL)
*Solidago canadensis*	Canada goldenrod	Analgesic (RT, FL), Emetic (RT, FL), Gastrointestinal (FL), Liver (FL), Sedative (RT)
Plants with no known medicinal use by Haudenosaunee^d^		
*Arabis glabra *(N)	Tower rockcress	None
*Myosotis scorpioides *(N)	True forget-me-not	None
*Silene virginica *(N)	Fire pink	None
*Hesperis matronalis *(I)	Dames rocket	None
*Rosa multiflora *(I)	Multiflora rose	None
*Silene latifolia *(I)	Bladder campion	None

^a ^Herrick 1977, 1995; Moerman 1996, 1998

^b ^Whole plant (PL), Flower (FL), Leaf (LV), Stem (ST), Root (RT)

^c ^Among approximately 150 species cited as the most powerful medicinal plants in Haudenosaunee culture (Herrick 1995) based on observed therapeutic and spiritual effects

^d ^Native (N), Introduced (I)

### Preparations of inoculums of microorganisms

Extracts were screened against two gram-negative bacteria (*Escherichia coli *[Presque Isle No.336] and *Salmonella typhimurium *[Presque Isle No.381]) and two gram-positive bacteria (*Staphylococcus aureus *[Presque Isle No.4651] and *Streptococcus lactis *[Presque Isle No.525]). *Escherichia coli *and *S. aureus *were maintained on Liquid Broth (LB) slant cultures (Krackeler Scientific, Albany, NY USA), and *S. typhimurium *and *S. lactis *were maintained on Brain Heart Infusion (BHI) slant cultures (Krackeler Scientific, Albany, NY USA). All slant cultures were kept at 37°C. Twenty-four hours before screening with disc diffusion or 96-well plate assays, replicate liquid cultures were started by placing a loopful of bacteria from the slant cultures into 10 mL of appropriate sterile media (*E. coli *and *S. aureus *in LB; *S. typhimurium *and *S. lactis *in BHI) and grown at 37°C. Immediately prior to all experiments, the turbidity of each liquid culture for use in the assays was adjusted to 0.5 McFarland Units (approx. 1 × 10^8 ^CFU/mL) using sterile LB or BHI.

### Disc diffusion assays

The sensitivity of different bacterial strains to the aqueous plant extracts was measured using a standard agar diffusion assay [20]. Sterile 100 × 15 mm Petri plates containing bacto-agar (Krackeler Scientific, Albany, NY USA) and either LB or BHI were prepared prior to beginning the assays, and 100 μL of liquid bacterial culture was spread onto plates with the appropriate media using sterile technique (*E. coli *and *S. aureus *on LB; *S. typhimurium *and *S. lactis *on BHI). Immediately prior to their placement on the plates, 10 μL of extract (100 mg/mL) was pipetted onto a 6 mm sterile filter paper disc. Each plate contained four paper discs; two discs contained extracts, one disc served as a negative control (10 μL sterile water), and one disc served as a positive control (10 μL of 10 mg/mL ampicillin). Each extract was tested on three replicate plates, and the plates were inverted and incubated at 37°C for 24 hours before being digitally photographed. Images were uploaded to a computer and zones of inhibition were calculated using ImageJ http://rsb.info.nih.gov/ij/. For each image, the pixel-to-centimeter relationship was calculated using a standard reference contained in the image, and the area and diameter of the zone of inhibition was measured directly. All zones of inhibition were circular.

### 96-well plate assays

Sixteen of the extracts were selected for use in the 96-well plate assay because they caused substantial zones of inhibition on one or more of the bacteria tested. Serial dilutions of each of these extracts were prepared using sterile water to produce final concentrations of 100% (not diluted), 50%, 25%, 12.5%, 6.25%, and 3.125%. In terms of fresh material to solvent, these dilutions represent concentrations of 100 mg/mL, 50 mg/mL, 25 mg/mL, 12.5 mg/mL, 6.25 mg/mL, and 3.125 mg/mL, respectively. The procedures used in this assay were similar to those used in another study where an indicator solution changes from clear to red in proportion to the degree of bacterial activity [21]. To start, the twelve-row by eight-column plate was visually divided into four sections of two columns each; one column was designated the "experimental" column and one was designated the "control". Each of these two-column sections was used to test a single extract as follows. Bacteria were prepared as described above (approx. 1 × 10^8 ^CFU/mL) to provide enough stock to fill all plates for all extracts tested (i.e., all of the tests involving a single bacteria species were performed on the same day). Then, 100 μL of liquid culture was placed into each cell of the experimental column along with 100 μL of each of the following solutions down the 8 rows: positive control (10 mg/mL ampicillin), negative control (sterile water), 100%, 50%, 25%, 12.5%, 6.25%, and 3.125% extract. The positive control cell (ampicillin) should have no bacterial activity and remain clear, whereas the negative control cell (water) should have bacterial activity and change to red, providing reference points to quantify the inhibitory effects of the extract dilutions. The control column only contained 100 μL of the positive control (ampicillin), negative control (water), and extraction dilution solutions (i.e., identical to the experimental column except no bacteria were present). Plates were prepared in triplicate such that each extract-bacteria combination was replicated across 96-well plates. Plates were covered and incubated at 37°C for 24 hours. After the 24-hour incubation, 40 μL of a 0.2 mg/mL *p*-iodonitrotetrazolium violet (INT) indicator solution was simultaneously added to every well and the plate was incubated at 37°C for an additional 30 minutes. The INT indicator solution changes from clear to red in the presence of bacterial activity and the degree of redness is a good measure of inhibitory effects [21]. Following the 30-minute incubation, plates were digitally photographed.

In order to assess the inhibitory effects of each of the extracts, images were uploaded to a computer and ImageJ http://rsbweb.nih.gov/ij/ was used to calculate the mean gray value by converting each pixel within the well to grayscale and then summing the gray values of all the pixels in the well and then dividing by the total number of pixels. ImageJ uses a standard formula to perform this conversion: gray = 0.299 red + 0.587 green + 0.114 blue. Then, the mean gray value of the cell containing the extract alone was subtracted from the cell containing the extract and bacteria combination to remove the effects of extract coloration on gray value. This difference was divided by the negative control (water) gray value to determine the fraction of bacteria alive in the experimental cell relative to the cell containing only water, and for simplicity of interpretation this quotient was subtracted from 1 to convert the fractions into a "proportion killed" as opposed to a "proportion alive". Although it is possible to split out the red values independent of the green and blue values and perform a similar analysis, preliminary experiments with a series of controlled-dye solutions showed that converting to gray values explained more of the observed variation in color (*Frey, unpublished data*).

### Statistical analyses

In the disc diffusion assays, the average size of the zone of inhibition (*N *= 3) is reported for those extracts that resulted in a consistent and noticeable kill zone compared to the diameter of the filter paper disc (6 mm). These extract-bacteria combinations were selected for use in the 96-well plate assays. For each extract-bacteria combination, a regression analysis was performed as follows [22]. We first transformed concentration values by taking the negative value of the base-10 logarithm for each. This generated an x-axis whereby the smallest value (-2) denoted the strongest concentration (100%), the largest value (-0.49) denoted the weakest concentration (3.125%), and the slopes of the relationship were negative. Then, we regressed the transformed concentration value on proportion killed for each extract-bacteria combination to estimate the slope and y-intercept of the observed relationship. In addition to determining the strength and explanatory power of this linear relationship, we calculated a conservative estimate of the x-intercept. To do this, we determined the 95% confidence limits of the observed regression coefficient and then used the upper 95% limit as well as the observed y-intercept to calculate a predicted x-intercept. In other words, we used the steepest of likely slopes to predict the largest concentration at which the extract would stop killing bacteria. Once we determined this x-intercept, we back-transformed it to values of mg/mL extract. The smallest directly observable MIC in our experiment is 3.125 mg/mL; however, the strength of the observed linear relationships in our study gives us a fair amount of confidence in our conservatively predicted estimate as well.

## Results

Of the 57 aqueous extracts, only 11 resulted in consistent inhibition zones against one or more of the bacteria tested in the disc diffusion assays (Table 2). There were a total of 16 extract-bacteria combinations that yielded inhibition zones. The average inhibition zone diameter of the extracts was substantially smaller (roughly one third) than that caused by ampicillin (10 mg/mL). Three of the extractions caused inhibition zones in more than one of the bacterial species tested (*A. millefolium *flower: *S. typhimurium *and *S. aureus*; *I. pandurata *leaf: *E. coli, S. typhimurium, S. aureus, S. lactis*; *R. multiflora *flower: *E. coli *and *S. typhimurium*). There was little consistency among extractions made from different parts of the same plant. In no case did all four extractions from a single species result in inhibition zones against the same bacterial species; in only three cases did extractions made from two different parts of the same plant result in inhibition zones against the same bacteria (*H. pilosella, I. pandurata*, and *R. multiflora*). One extract caused a *S. lactis *inhibition zone, three extracts caused an *E. coli *inhibition zone, five extracts caused a *S. typhimurium *inhibition zone, and seven extracts caused a *S. aureus *inhibition zone.

**Table 2:** Mean diameter of inhibition zones (mm) where bacterial growth was inhibited by plant extracts.

		**Mean inhibition zone diameter**^**a**^			
		Gram-negative organisms		Gram-positive organisms	
			
Plant Species	**Portion**^**b**^	*E. coli *(PI No.336)	*S. typhimurium *(PI No.381)	*S. aureus *(PI No.4651)	*S. lactis *(PI No.525)
Haudenosaunee medicinal plants					
*A. millefolium*	FL	------	8.8	9.6	------
					
*H. pilosella*	FL	------	------	8.1	------
	LV	------	------	9.0	------
	ST	------	9.1	------	------
					
*I. pandurata*	FL	------	------	7.3	------
	LV	10.2	11.1	15.8	12.3
					
*S. canadensis*	LV	------	------	9.3	------
					
Plants with no known medicinal use by Haudenosaunee					
*S. virginica*	LV	------	------	7.9	------
					
*H. matronalis*	ST	------	11.1	------	------
					
*R. multiflora*	FL	9.0	8.1	------	------
	LV	7.6	------	------	------

Ampicillin (10 mg/mL)		35.0	29.6	37.9	33.2
Water		------	------	------	------

^a ^Values represent the mean of three replicates; the pooled SD of all means = 0.60 mm.

^b ^10 μL of each extract (100 mg/mL fresh water extraction) was used for each plant portion; FL, LV, and ST denote flowers, leaves, and stems, respectively.

**Figure 1:**
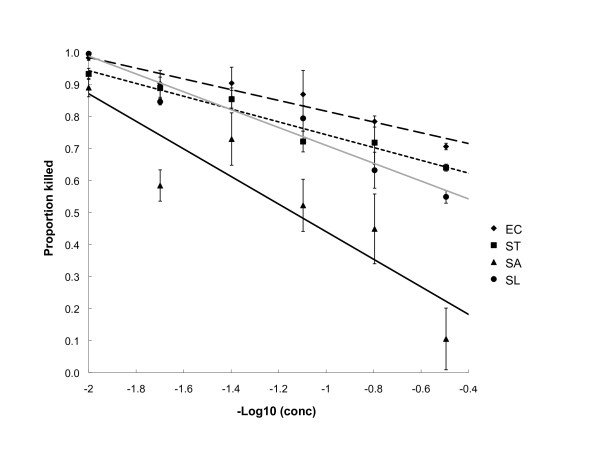
**Antibacterial activity of I. pandurata**. Results of 96-well plate assay for *I. pandurata *leaf extracts on all four bacteria. The average proportion (± 1 standard error; *n *= 3) of bacteria killed by each extract dilution is shown with a fitted linear regression line (see Table 2). To facilitate analyses, the extract concentration has been transformed by taking the negative base-10 logarithm (strongest to weakest dilution left to right). Diamonds and the dashed line denote *E. coli *(EC), squares and the dotted line denote *S. typhimurium *(ST), triangles and the solid black line denote *S. aureus *(SA), and circles and the solid grey line denote *S. lactis *(SL).

The serial dilution assay suggested that the inhibitory effects of the 16 extract-bacteria combinations revealed in the disc diffusion assay were dose dependent (Table 3). In all cases, concentration explained a significant amount of variation in the proportion of bacteria killed as measured using the INT indicator solution (average R^2 ^value = 74%; negative slope in all cases). Figure 1 shows a graphical example of the statistical relationships. The directly observable MIC for *S. aureus *in most cases was 12.5 mg/mL, except for the *S. canadensis *leaf extraction where the MIC was 3.125 mg/mL. All other extract-bacteria combinations had directly observable MICs of 3.125 mg/mL.

**Table 3:** Regression statistics, observed MIC (mg/mL) values and predicted MIC (mg/mL) values for all plant-extract and bacteria combinations with demonstrated inhibition in the disc diffusion assays.

**Extract**^**a**^	Bacteria	***R***^***2***^	**B (SE)**^**b**^	**MIC (obs)**^**c**^	**MIC (pred)**^**d**^
Haudenosaunee medicinal plants					
Am-FL	*S. typhimurium*	0.492	-0.112 (0.036)**	3.125	0.010
Am-FL	*S. aureus*	0.804	-0.667 (0.104)***	12.5^e^	19.1
Hp-FL	*S. aureus*	0.893	-0.818 (0.107)***	12.5^e^	36.0
Hp-LV	*S. aureus*	0.949	-1.196 (0.104)***	12.5^e^	23.6
Hp-ST	*S. typhimurium*	0.626	-0.108 (0.021)***	3.125	0.001
Ip-FL	*S. aureus*	0.791	-0.704 (0.114)***	12.5^e^	21.1
Ip-LV	*E. coli*	0.674	-0.169 (0.029)***	3.125	0.006
Ip-LV	*S. typhimurium*	0.853	-0.200 (0.026)***	3.125	0.023
Ip-LV	*S. aureus*	0.682	-0.425 (0.072)***	3.125	3.1
Ip-LV	*S. lactis*	0.859	-0.285 (0.030)***	3.125	0.1
Sc-LV	*S. aureus*	0.864	-0.640 (0.064)***	3.125	3.6
					
Plants with no known medicinal use by Haudenosaunee					
Sv-LV	*S. aureus*	0.715	-0.221 (0.026)***	12.5^e^	10.1
Hm-ST	*S. typhimurium*	0.476	-0.134 (0.035)**	3.125	0.002
Rm-FL	*E. coli*	0.820	-0.236 (0.028)***	3.125	0.036
Rm-FL	*S. typhimurium*	0.696	-0.108 (0.021)***	3.125	0.053
Rm-LV	*E. coli*	0.714	-0.155 (0.024)***	3.125	0.004

^a ^*A. millefolium *(Am), *H. matronalis *(Hm), *H. pilosella *(Hp), *I. pandurata *(Ip), *R. multiflora *(Rm), *S. virginica *(Sv), *S. canadensis *(Sc); Flower (FL), Leaf (LV), Stem (ST).

^b ^Unstandardized regression coefficient and standard error. ** indicates *P *< 0.01; *** indicates *P *< 0.001. There were three replicates for each dilution series.

^c ^MIC (mg/mL) observed in serial dilution assay.

^d ^MIC (mg/mL) calculated utilizing 95% confidence limit of regression slope to conservatively predict the concentration at which the proportion of bacteria killed goes to zero.

^e ^Because the observed proportion of bacteria killed went to zero by the 6.25% dilution (6.25 mg/mL), the regression statistics were calculated using only the first four points of the dilution series.

We used the 95% confidence interval associated with the slope of each regression analyses to conservatively predict the MIC drawing on the linear trend observed in the serial dilution assay. Although by no means conclusive, this analysis gives insight into the relative efficacy of the extractions. The conservative nature of these estimates is illustrated by most of the *S. aureus *results where the predicted MIC is greater than the observed MIC. This analysis suggests that particular extract-bacteria combinations may result in MICs on the order of tens of μg/mL (*H. matronalis *stem - *S. typhimurium*; *H. pilosella *leaf - *S. typhimurium*; *I. pandurata *leaf - *E. coli*; *R. multiflora *leaf - *E. coli*) or hundreds of μg/mL (*A. millefolium *flower - *S. typhimurium*; *I. pandurata *leaf - *S. typhimurium*; *R. multiflora *flower - *E. coli *and *S. typhimurium*).

## Discussion

Traditional Haudenosaunee medicine involves a number of plant species native to NY. We selected a small subset of these plants and attempted to determine whether they exhibited antibacterial properties as measured through two different assays. In the discussion that follows, we first interpret these data with respect to our two main hypotheses and then turn to a discussion of those species that might indeed exhibit antibacterial properties. Before doing so, there are two important caveats of our study to highlight. First, although the medicinal uses of these plants were in the form of water-based poultices, decoctions, and infusions [6,7], our extraction method most certainly does not capture the nuances of the actual preparations used. Second, we have no evidence that the bacteria used in this study (e.g., *S. typhimurium*) were indeed the causal agents underlying the symptoms associated with the use of these plants (e.g., infusion of smashed *A. millefolium *plants to treat diarrhea).

Our data are mixed with respect to the hypothesis that a correlation exists between the use of Haudenosaunee medicinal plants and antibacterial properties. Our analyses failed to find any evidence of antibacterial activity for three plants used in Haudenosaunee medicines (*A. syriaca, M. neglecta*, and *R. acris*) to treat broad-spectrum gastrointestinal ailments and as an antidiarrheal [6,7]. However, extractions from four plants exhibited antibacterial properties as expected (*A. millefolium, I. pandurata, H. pilosella*, and *S. canadensis*), and it is interesting to note that neither of the two plant species used to treat non-bacterial symptoms (*C. racemosa *and *L. digitatum*), and only one of the native plant species without an ascribed medicinal use did so (*S. virginica*). We also hypothesized that if these plants had antibacterial properties, we would likely observe stronger effects in moderately virulent gram-negative and gram-positive bacteria (*S. typhimurium *and *S. aureus*) than in mostly avirluent gram-negative and gram-positive bacteria (*E. coli *and *S. lactis*). The data are in line with this prediction as 12/16 (75%) of the extracts causing inhibition zones did so against the moderately virulent bacteria. Taken together, these data suggest that although there is not complete concordance between ascribed use and antibacterial activity in the laboratory, the selection and use of these plants to treat disease was likely non-random.

Yarrow (*A. millefolium*) is recognized as a powerful medicinal plant in Haudenosaunee culture [6,7], is widely distributed and has been used medicinally by a number of other cultures for thousands of years [23]. In our study, *A. millefolium *showed antibacterial activity against *S. typhimurium *and *S. aureus *with predicted MICs on the order of 10 s of μg/mL or 10 s of mg/mL, respectively. A number of studies have investigated the antibacterial properties of this species [24-28] and found similar results to those presented here. One difference, however, is that two studies found that ether-hexane-methanol extracts of Yarrow caused inhibition zones against *E. coli *in disc diffusion assays [27,28], whereas our study with aqueous extracts of flower, leaves, roots, and shoots and a separate study of essential oil and methanolic extracts [26] did not. These differing results could be due to the different extraction methods used or regional variation in the chemical constituents of the plants. It is well known that Yarrow represents a diverse, polyploid complex that is probably composed of dozens of species with varying biochemical compositions [29]. The biochemical diversity of this complex has been fairly well described [30,31], and it has been hypothesized that phenolic compounds such as flavonoids and phenolcarbonic acids may underlie the observed antimicrobial activity [27,28,31]. A number of other studies have found additional therapeutic properties including anti-inflammatory [32], antioxidant [26], and antinociceptive [33] properties. Moreover, there is growing evidence that both flavonoids and sesquiterpenoids have anti-proliferative effects against mouse P-388 leukemia cells [34] and cervix epithelial adenocarcinoma (HeLa), breast epithelial adenocarcinoma (MCF-7) and skin epidermoid carcinoma (A431) cells [35]. Clearly, Yarrow holds great potential as a source of many new drugs.

Man-of-the-Earth (*I. pandurata*) is also recognized as a powerful medicinal plant in Haudenosaunee culture [6,7], but is far less studied than Yarrow. In fact, we know of no previous investigations into the possible therapeutic properties of this species. Our results show that aqueous leaf extracts are strong inhibitors of all species of bacteria used in this study with predicted MICs of 1-10 s of μg/mL against *E. coli *and *S. typhimurium *and 100-1000 s of μg/mL against *S. aureus *and *S. lactis*. There are a few other investigations of other morning glory species in the literature showing antibacterial properties of *I. batatas *[36], *I. murucoides *[37] and *I. stans *[38]. In addition, there are studies demonstrating antifungal properties of undescribed *Ipomoea *species [39], antinociceptive properties of *I. pes-caprae *[40] and *I. cairica *[41], anti-inflammatory and anti-spasmodic activity of *I. imperati *[42], central nervous system depressant activity of *I. stans *[43] as well as cytotoxic activity [38], and antimycobacterial and cytotoxic activity of *I. tyrianthina *[44,45].

Mouseear Hawkweed (*H. pilosella*) and Canada goldenrod (*S. canadensis*) were both used by Haudenosaunee peoples to treat gastrointestinal ailments and diarrhea [6,7]. Our results show that mouseear hawkweed may weakly inhibit *S. aureus *(predicted MICs of 10 s of mg/mL) and more strongly inhibit *S. typhimurium *(predicted MIC of 1-10 s of μg/mL). These results are consistent with the results of a separate study that utilized methanol, dichloromethane, ethyl acetate, and dichloromethane:methanol (9:1) extractions [46], and with a study that utilized methanol and aqueous extracts [47]; however, our aqueous extractions were not effective against *E. coli *as in the other extractions of these two studies. We are aware of only two other investigations of this species, finding that flavonoids from *H. pilosella *have antioxidant activity [46,48]. Canada goldenrod only inhibited *S. aureus *in our study at a relatively weak predicted MIC (1-10 s of mg/mL). Although we know of no other studies that have investigated the antimicrobial properties of this species, methanol extractions of root tissue show moderate antioxidant activity [49], and two studies have documented the antifungal properties of the essential oil of *S. chilensis *[50] and the anti-inflammatory properties of ethanol extracts of *S. chilensis *[51].

It was somewhat surprising that Fire Pink (*S. virginica*) weakly inhibited the growth of *S. aureus *(predicted MIC of 10 s of mg/mL). The *Silene *complex is well known for phytoecdysteroids [52,53], but this is the first report of possible antibacterial properties. Likewise, it was surprising that Dames Rocket (*H. matronalis*) was strongly effective against *S. typhimurium *(predicted MIC of 1-10 s of μg/mL) because we know of no other studies showing any therapeutic properties of this genus. There are a number of studies demonstrating the nutritional, anti-inflammatory, analgesic, and anti-oxidant properties of Multiflora Rose (*R. multiflora*) hips and other members of the genus [54,55]. A study of wild rose hips in Canada found some evidence for weak anti-microbial activity against yeast and gram-positive bacteria [56]. However, our results show that flower and leaf aqueous extracts are effective against gram-negative bacteria (*E. coli *and *S. typhimurium*) at predicted MICs on the order of 10 s of μg/mL.

## Conclusions

Growing antibiotic resistance among human pathogens [57-59] and new data showing that antibiotic-resistant *E. coli *can protect antibiotic-sensitive *S. typhimurium *without gene transfer [60], emphasize the importance of finding new antibacterial molecules. Our data suggest that investigating traditional Haudenosaunee medicinal plants may yield promising new leads. The degree of concordance between traditional use and observed antibacterial properties suggest that there may be some truth to these remedies. In particular, our results suggest that *A. millefolium, H. pilosella, I. pandurata*, and *S. canadensis *warrant further study, as does the previously undocumented *H. matronalis*, especially in the context of *S. typhimurium*. Elucidating the mode of action behind these observed antibacterial properties, as well as exploring other pharmacological activities is currently underway in our lab.

## Competing interests

The authors declare that they have no competing interests.

## Authors' contributions

FMF and RM conceived of, designed, and conducted this study. FMF analyzed the data and wrote the manuscript. Both authors evaluated the results and corrected the manuscript for publication. Both authors read and approved of the final manuscript.

## Pre-publication history

The pre-publication history for this paper can be accessed here:

http://www.biomedcentral.com/1472-6882/10/64/prepub
